# Exposure to 6-PPD Quinone Disrupts Adsorption and Catabolism of Leucine and Causes Mitochondrial Dysfunction in *Caenorhabditis elegans*

**DOI:** 10.3390/toxics13070544

**Published:** 2025-06-28

**Authors:** Wei Wang, Yunhui Li, Dayong Wang

**Affiliations:** 1 Shenzhen Ruipuxun Academy for Stem Cell & Regenerative Medicine, Shenzhen 518122, China; 2 School of Public Health, Southeast University, Nanjing 210009, China; 3 Medical School, Southeast University, Nanjing 210009, China

**Keywords:** leucine, adsorption, catabolism, mitochondrial function, pharmacological treatment, nematodes

## Abstract

6-PPD quinone (6-PPDQ) is a derivative from 6-PPD, an antioxidant added in tires. Leucine is an important amino acid that needs to be obtained from the diet. In *Caenorhabditis elegans*, we examined the effect of 6-PPDQ exposure at environmentally relevant concentrations (ERCs) on the content of leucine and underlying mechanisms. In nematodes, 0.1–10 μg/L of 6-PPDQ decreased leucine content. The expression of the *aat-1*-encoding amino acid transmembrane transporter was decreased by 0.1–10 μg/L of 6-PPDQ, and leucine content was reduced by *aat-1* RNAi. Meanwhile, the expression of *bcat-1*-encoding branched-chain amino acid transferase was increased by 0.1–10 μg/L of 6-PPDQ, and leucine content was increased by *bcat-1* RNAi. Additionally, the expressions of *dbt-1* and *ivd-1* encoding two enzyme genes governing NADH and FADH_2_ generations were decreased by 0.1–10 μg/L of 6-PPDQ, and their expressions in 6-PPDQ exposed nematodes were increased by *bcat-1* RNAi. After 6-PPDQ exposure, NADH content was reduced by *dbt-1* RNAi, and FADH_2_ content was reduced by *ivd-1* RNAi. Moreover, 6-PPDQ-induced mitochondrial dysfunction and other aspects of toxicity (such as intestinal ROS generation and lipofuscin accumulation, inhibited locomotion, and reduced brood size) were suppressed by *bcat-1* RNAi and strengthened by *dbt-1* and *ivd-1* RNAi. The 6-PPDQ-induced toxicity and the decrease in *dbt-1* and *ivd-1* expressions could be inhibited by following leucine (5 mM) treatment. Our results demonstrate the important association of leucine adsorption and catabolism with 6-PPDQ toxicity induction.

## 1. Introduction

*N*′-(1,3-Dimethylbutyl)-*N*′-phenyl-p-phenylenediamine (6-PPD) is widely applied in tires to delay their oxidation [1]. 6-PPDQ was initially observed to induce the lethality of salmon coho [2]. After reacting with ozone through different pathways, 6-PPDQ can be formed from 6-PPD [3]. In environments including water, soil, snow, sediment, and dust, 6-PPDQ can be detected [4,5,6,7,8]. Environmentally relevant concentrations (ERCs) of 6-PPDQ have been found in the range of ng/L to tens of μg/L [9,10,11,12]. Besides the induction of acute lethality [2,13], 6-PPDQ also causes toxicity on development, behavior, and reproduction and disruption in metabolisms and the intestinal microbial community in environmental animals [14,15,16,17]. In mammals, exposure to 6-PPDQ can further induce impairment on sperm quality [18], as well as the intestine [19] and the liver [20]. More recently, it was observed that mitochondrial dysfunction can be caused by 6-PPDQ exposure [21,22,23,24].

*Caenorhabditis elegans* has a small size and well-described genetic and molecular backgrounds. After exposure to pollutants, *C. elegans* can exhibit high sensitivity [25,26,27]. Due to these properties, it has been used to assess pollutant toxicity at ERCs [28,29,30]. In *C. elegans*, 6-PPDQ can result in multiple toxicities, such as neurotoxicity and intestinal toxicity [31,32]. Additionally, some metabolisms, including those of glycogen and glucose, are disrupted by 6-PPDQ exposure [33,34]. Moreover, 6-PPDQ exposure induces mitochondrial dysfunction, which is associated with both damage to complexes (such as complexes I and II) and suppression in mitochondrial UPR [35,36].

**Figure 1:**
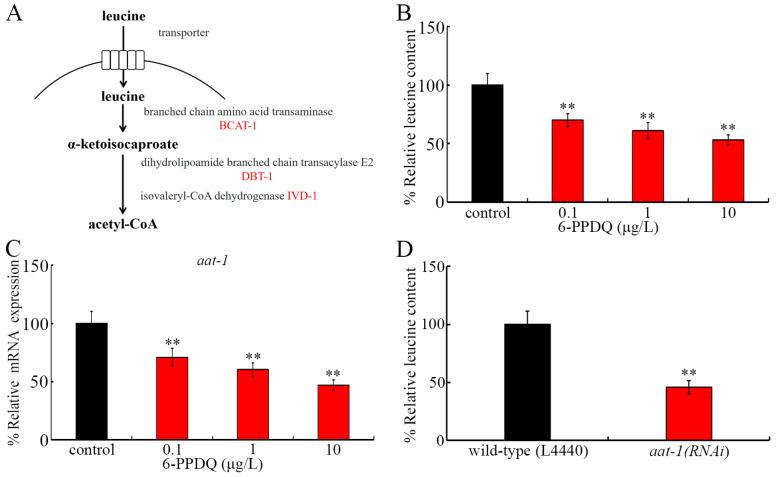
The effect of 6-PPDQ exposure on leucine content and adsorption. (**A**) A diagram showing leucine adsorption and catabolism in *C. elegans*. (**B**) The effect of 6-PPDQ exposure on leucine content. ** *p* < 0.01 vs. control. (**C**) The effect of 6-PPDQ exposure on intestinal *aat-1* expression. ** *p* < 0.01 vs. control. Thirty intact intestines were isolated for qRT-PCR analysis. (**D**) The effect of RNAi of *aat-1* on leucine content in 6-PPDQ-exposed nematodes. The exposure concentration of 6-PPDQ was 10 μg/L. ** *p* < 0.01 vs. wild-type (L4440).

Leucine cannot be synthesized de novo [37]. Leucine plays important functions in modulating some physiological processes, including energy balance and protein synthesis [38]. Some solute carriers function to facilitate the transmembrane transport of amino acids from the intestinal lumen to intestinal cells [39,40], and *C. elegans* AAT-1 is the amino acid transmembrane transporter expressed in the intestine. Once inside the cells, leucine is catalyzed by branched-chain amino acid transferase (BCAT-1) into α-ketoisocaproate (Figure 1A) [41,42]. After this, NADH and FADH_2_ can be generated from the intermediates through the actions of dihydrolipoamide branched-chain transacylase E2 DBT-1 and isovaleryl-CoA dehydrogenase IVD-1 (Figure 1A) [42,43]. We assumed that exposure to 6-PPDQ may disrupt the adsorption and catabolism of leucine, which is associated with the induction of mitochondrial dysfunction. Thus, we first aimed to explore whether exposure to 6-PPDQ disrupts leucine adsorption and catabolism and its association with mitochondrial dysfunction in nematodes. Additionally, the pharmacological effect of leucine treatment against 6-PPDQ damage on mitochondrial function was determined. The results suggested an important link between disruption in leucine adsorption and catabolism and 6-PPDQ toxicity induction.

## 2. Materials and Methods

### 2.1. Animal Maintenance

Animals were normally cultured on nematode growth medium (NGM) plates fed with *E. coli* OP50 [44]. The used strain was wild-type N2. Eggs were prepared by treating hermaphrodite nematodes using an alkaline lysis solution [45] and were further cultured on new NGM plate to obtain the L1 larval population.

### 2.2. 6-PPDQ Exposure

The 6-PPDQ was purchased from Toronto Research Chemicals Co. Its working solutions (0.1, 1, and 10 μg/L) were selected based on reported ERCs of 6-PPDQ in the environment [3,11]. The L1 larvae were exposed to 6-PPDQ until they reached adult day-3 (approximately 6.5 days) [46]. The 6-PPDQ solutions were refreshed daily.

### 2.3. Leucine Content Assay

Approximately 0.2 g of nematodes were harvested from each group and homogenized on ice in a PBS buffer. The homogenate was centrifuged for 10 min at 12,000× *g*. The leucine content was measured using a leucine determination kit (Wuhan Mosher Biotechnology Co., Wuhan, China). Supernatants and an enzyme-labeled reagent were added to the enzyme-labeled plates to incubate at 37 °C for 1 h. Subsequently, 50 μL of chromogenic reagents A and B were added. The absorbance was measured at 450 nm. The experiments were repeated three times.

### 2.4. Transcriptional Expression

The total nematode RNAs were extracted from approximately 500 animals using Trizol, and their quality was evaluated using an A260/280 ratio. The total RNAs were reverse transcribed to obtain cDNA. Gene expression was determined in the StepOnePlus™ real-time PCR system (Applied Biosystems, Carlsbad, CA, USA) using the SYBR Green qRT-PCR master mix (Takara, Kusatsu, Japan) and analyzed using the comparative cycle threshold method. *tba-1* was used as the reference gene [47]. The experiments were repeated three times. Information on the primers is available in Table S1.

### 2.5. NADH and FADH_2_ Contents

The NADH content was measured based on the kit instructions (Yfxbio Biotech, Nanjing, China). Approximately 0.2 g of nematodes were harvested from each group and homogenized in 400 μL of the extraction buffer. After centrifugation for 10 min at 12,000× *g*, the supernatant was incubated with hydrolyzed NAD^+^ at 60 °C, and working reagents were further added for absorbance measurement at 450 nm. The NADH concentrations were assessed based on a prepared standard curve. The FADH_2_ content was measured using a FADH_2_ ELISA kit (Shanghai Yansheng Biochemical Reagent Co., Shanghai, China). Supernatant and detection antibodies were added to the enzyme-labeled plates to incubate at 37 °C for 1 h. After this, substrate reagents A and B were added, gently mixed, and incubated at 37 °C for 15 min. The absorbance was measured at 450 nm. The experiments were repeated three times.

### 2.6. Mitochondrial Function

The endpoints of oxygen consumption rate and ATP content reflect the mitochondrial function [48]. Approximately 0.1 g of nematodes were collected and homogenized in pre-cooled lysate. Mitochondria were prepared using the differential centrifugation method. For the oxygen consumption assay, 100 μL of mitochondria were incubated together with 4 μL of the BBoxiProbe R01 oxygen fluorescence probe (Sangon, Shanghai, China). The absorbance was measured at 468 nm at 5 min intervals (for 30 min). For the ATP content assay, based on the ATP kit protocol (Sangon, Shanghai, China), the homogenate was centrifuged at 12,000× *g* for 5 min. The supernatants or standard liquids were used for the ATP content assay. The absorbance was measured at 340 nm. The ATP detection working solution was applied to evaluate the background ATP. The experiments were repeated three times.

### 2.7. Toxicity Assessments

Intestinal reactive oxygen species (ROS) generation and lipofuscin accumulation were used to reflect intestinal toxicity. To assess ROS generation, nematodes were labelled using 1 mM of CM-H_2_DCFDA for 3 h [49]. After washing using M9 buffer, the nematodes were observed at 510 nm of the emission filter and at 488 nm of the excitation wavelength. Intestinal ROS fluorescence signals were analyzed under upright fluorescence microscopy (AX10, Zeiss, Oberkochen, Germany). To assess lipofuscin accumulation, nematodes were analyzed under a DAPI filter using a fluorescence microscope [50]. The fluorescence intensity of ROS signals and lipofuscin accumulation were quantified in images (magnification, 20×) using the ImageJ V1.8.0.112. Fifty animals were examined per treatment. The experiments were repeated three times.

Head thrash and body bend reflect the locomotion behavior. Their frequencies were recorded in one minute or in 20 s intervals, respectively [51]. A change in the bending direction at the body mid-region is considered to be a head thrash. A change in the posterior bulb direction is considered to be a body bend. Fifty nematodes were analyzed per treatment. The experiments were repeated three times.

Brood size reflects the reproductive capacity. Brood size indicates the total number of offspring produced by each nematode, which was measured until the nematode ceased egg-laying [52]. Thirty nematodes were analyzed per treatment. The experiments were repeated three times.

### 2.8. RNA Interference (RNAi)

To perform the RNAi of certain genes, the nematodes were cultured on NGM plates fed with *E. coli* HT115 bacteria expressing corresponding double-stranded RNA [53]. The RNAi bacteria were prepared in a Luria broth containing 100 μg/mL of ampicillin and 100 μg/mL of tetracycline. The offspring of the nematodes cultured on RNAi plates were used for 6-PPDQ exposure. L4440 (empty vector) was used as the control [54]. RNAi efficiency is shown in Figure S1.

### 2.9. Pharmacological Treatment

Following 6-PPDQ exposure (10 μg/L) until adult day 3, the nematodes were washed with K buffer three times. After that, one subgroup was maintained on NGM medium, and another group was transferred into 5 mM leucine to be treated for 24 h at room temperature in darkness [55]. The experiments were conducted in triplicate.

### 2.10. Data Analysis

Data are presented the mean ± standard deviation (SD). Data were all continuous and passed the normality test and the homogeneity test of variance in SPSS 26.0. A one-way analysis of variance (ANOVA) was used to examine differences between different groups. For multiple factor comparison, two-way ANOVA analysis followed by a post-hoc test was employed. A *p*-value of < 0.01 (^**^) was considered to be statistically significant.

## 3. Results

### 3.1. 6-PPDQ Reduced Leucine Content

The leucine content in wild-type nematodes could be reduced by 0.1–10 μg/L of 6-PPDQ (Figure 1B). The reduction in leucine content was concentration-dependent in nematodes exposed to 0.1–10 μg/L of 6-PPDQ (Figure 1B). The leucine content was reduced by 30%, 39%, and 47%, respectively, after exposure to 0.1–10 μg/L of 6-PPDQ (Figure 1B).

### 3.2. Exposure to 6-PPDQ Inhibited Leucine Adsorption

After 0.1–10 μg/L 6-PPDQ exposure in wild-type nematodes, intestinal *aat-1* expression was decreased (Figure 1C). Meanwhile, after *aat-1* RNAi, the leucine content could be decreased by 54% in 6-PPDQ-exposed nematodes (Figure 1D). Therefore, leucine adsorption was inhibited after 6-PPDQ exposure.

### 3.3. 6-PPDQ Accelerated 6-PPDQ Catabolism

**Figure 2:**
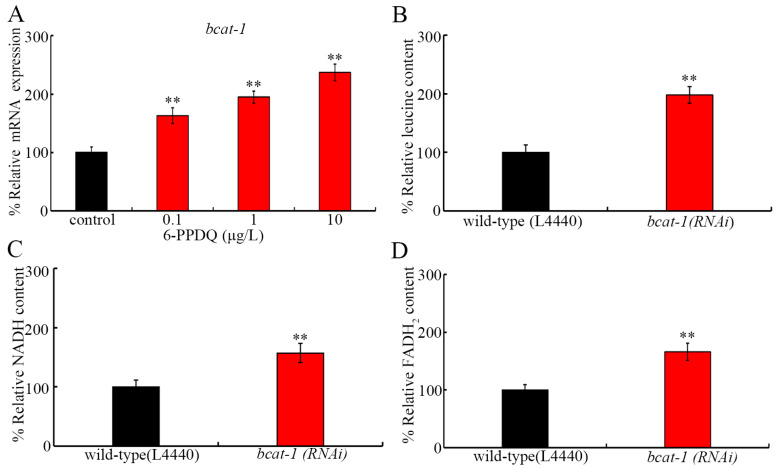
Effect of 6-PPDQ exposure on leucine catabolism. (**A**) Effect of 6-PPDQ exposure on *bcat-1* expression. ** *p* < 0.01 vs. control. (**B**) Effect of RNAi of *bcat-1* on leucine content in 6-PPDQ-exposed nematodes. Exposure concentration of 6-PPDQ was 10 μg/L. ** *p* < 0.01 vs. wild-type (L4440). (**C**) Effect of RNAi of *bcat-1* on NADH content in 6-PPDQ-exposed nematodes. Exposure concentration of 6-PPDQ was 10 μg/L. ** *p* < 0.01 vs. wild-type (L4440). (**D**) Effect of RNAi of *bcat-1* on FADH_2_ content in 6-PPDQ-exposed nematodes. Exposure concentration of 6-PPDQ was 10 μg/L. ** *p* < 0.01 vs. wild-type (L4440).

After 6-PPDQ exposure in wild-type nematodes, *bcat-1* expression was further increased (Figure 2A). Meanwhile, after 6-PPDQ exposure, the leucine content was increased by *bcat-1* RNAi (Figure 2B). Moreover, after 6-PPDQ exposure, we observed an increase in NADH and FADH_2_ contents in *bcat-1 (RNAi)* nematodes compared with the wild-type (L4440) (Figure 2C,D). That is, leucine catabolism could be enhanced by 6-PPDQ exposure, which was associated with a reduction in NADH and FADH_2_ contents.

### 3.4. 6-PPDQ Affected Expressions of dbt-1 and ivd-1 and Their Functions

**Figure 3:**
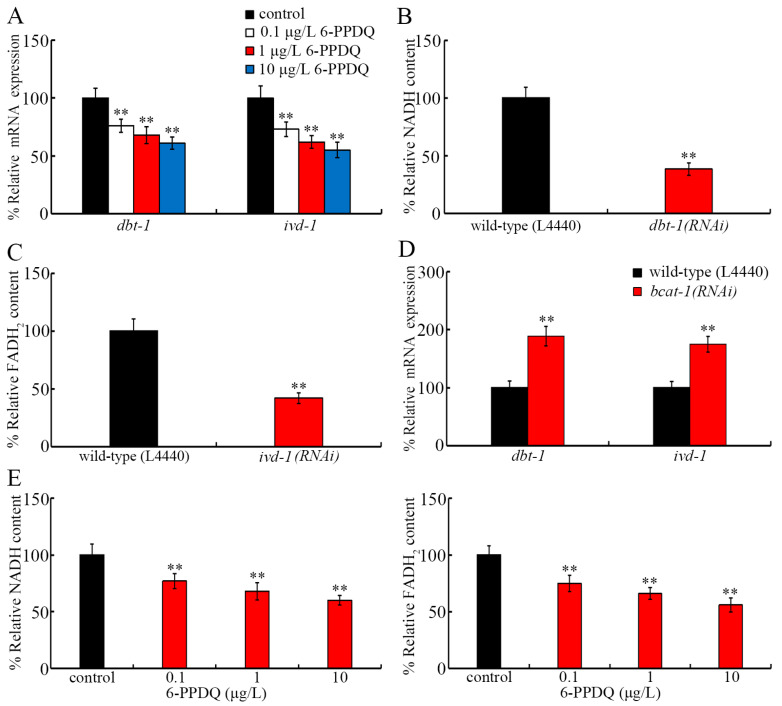
Effect of 6-PPDQ exposure on expressions of *dbt-1* and *ivd-1* and their functions. (**A**) Effect of 6-PPDQ exposure on expressions of *dbt-1* and *ivd-1*. ** *p* < 0.01 vs. control. (**B**) Effect of RNAi of *dbt-1* on NADH content in 6-PPDQ-exposed nematodes. Exposure concentration of 6-PPDQ was 10 μg/L. ** *p* < 0.01 vs. wild-type (L4440). (**C**) Effect of RNAi of *ivd-1* on FADH_2_ content in 6-PPDQ-exposed nematodes. Exposure concentration of 6-PPDQ was 10 μg/L. ** *p* < 0.01 vs. wild-type (L4440). (**D**) Effect of RNAi of *bcat-1* on expressions of *dbt-1* and *ivd-1* in 6-PPDQ-exposed nematodes. Exposure concentration of 6-PPDQ was 10 μg/L. ** *p* < 0.01 vs. wild-type (L4440). (**E**) Effect of 6-PPDQ exposure on NADH and FADH_2_ contents. ** *p* < 0.01 vs. control.

Considering the possible important role of DBT-1 and IVD-1 for the generation of NADH and FADH_2_ [42,43], we next investigated the effect of 6-PPDQ on the expressions of *dbt-1* and *ivd-1*. The expressions of *dbt-1* and *ivd-1* were decreased by 0.1–10 μg/L of 6-PPDQ in wild-type nematodes (Figure 3A). After 6-PPDQ exposure, the NADH content could be reduced by RNAi of *dbt-1* (Figure 3B), and the FADH_2_ content could be reduced by the RNAi of *ivd-1* (Figure 3C). Moreover, after 6-PPDQ exposure, *dbt-1* and *ivd-1* expressions were increased by *bcat-1* RNAi (Figure 3D). In 6-PPDQ-exposed wild-type nematodes, the NADH and FADH_2_ contents showed a tendency towards reduction (Figure 3E).

### 3.5. RNAi of bcat-1, dbt-1, and ivd-1 Affected 6-PPDQ-Induced Mitochondrial Dysfunction

**Figure 4:**
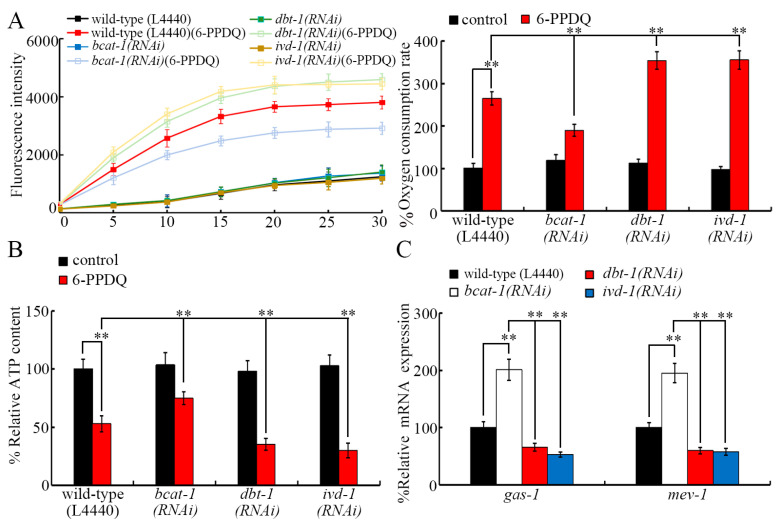
Effect of RNAi of *bcat-1*, *dbt-1*, and *ivd-1* on oxygen consumption rate (**A**), ATP content (**B**), and expressions of *gas-1* and *mev-1* (**C**) in 6-PPDQ-exposed nematodes. Exposure concentration of 6-PPDQ was 10 μg/L. ** *p* < 0.01.

After 6-PPDQ exposure, mitochondrial dysfunction was induced [56]. The 6-PPDQ-induced increase in the oxygen consumption rate was inhibited by the RNAi of *bcat-1* and was enhanced by the RNAi of *dbt-1* and *ivd-1* (Figure 4A). The 6-PPDQ-caused decrease in the ATP content was suppressed by the RNAi of *bcat-1* and strengthened by the RNAi of *dbt-1* and *ivd-1* (Figure 4B). GAS-1 (complex I component) and MEV-1 (complex II component) were involved in controlling 6-PPDQ-induced mitochondrial dysfunction [35]. Moreover, after 6-PPDQ exposure, *gas-1* and *mev-1* expressions were increased by *bcat-1* RNAi and decreased by *dbt-1* and *ivd-1* RNAi (Figure 4C).

### 3.6. RNAi of aat-1, bcat-1, dbt-1, and ivd-1 Affected Induction of 6-PPDQ Toxicity

**Figure 5:**
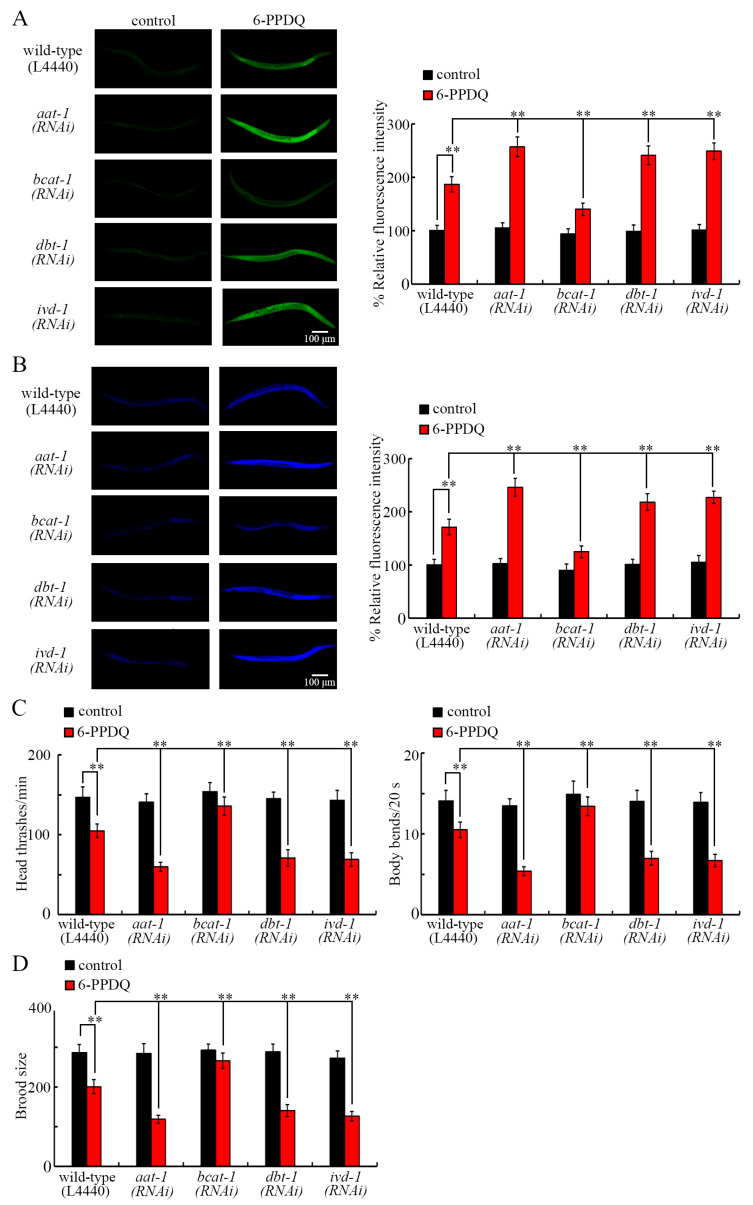
Effect of RNAi of *aat-1*, *bcat-1*, *dbt-1*, and *ivd-1* on 6-PPDQ toxicity in inducing intestinal ROS generation (**A**), causing intestinal lipofuscin accumulation (**B**), decreasing locomotion (**C**), and reducing brood size (**D**). Exposure concentration of 6-PPDQ was 10 μg/L. ** *p* < 0.01.

We further investigated the effect of *aat-1*, *bcat-1*, *dbt-1*, and *ivd-1* RNAi on other 6-PPDQ-induced aspects of toxicity. Intestinal toxicity could be induced by 6-PPDQ, reflected by intestinal ROS generation and lipofuscin accumulation [50]. After 6-PPDQ exposure, induced intestinal ROS generation and lipofuscin accumulation were enhanced by the RNAi of *aat-1*, *dbt-1*, and *ivd-1*, and inhibited by the RNAi of *bcat-1* (Figure 5A,B). Exposure to 6-PPDQ also caused neurotoxicity reflected by inhibited locomotion [50] and reproductive toxicity reflected by reduced brood size [32], which were strengthened by *aat-1*, *dbt-1*, and *ivd-1* RNAi and suppressed by *bcat-1* RNAi (Figure 5C,D).

### 3.7. Beneficial Effect of Leucine Treatment Against 6-PPDQ-Induced Mitochondrial Dysfunction

**Figure 6:**
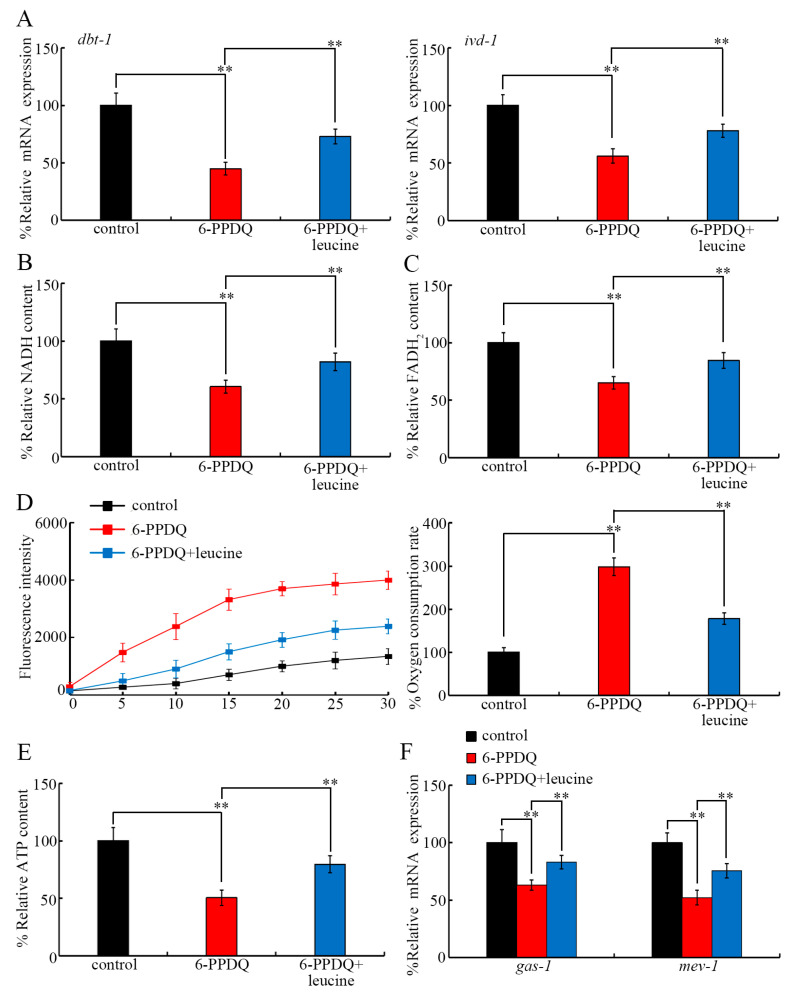
Effect of treatment with leucine (5 mM) on toxicity of 6-PPDQ (10 μg/L) in affecting expressions of *dbt-1* and *ivd-1* (**A**), NADH content (**B**), FADH_2_ content (**C**), oxygen consumption rate (**D**), ATP content (**E**), and expressions of *gas-1* and *mev-1* (**F**). ** *p* < 0.01.

After exposure to 6-PPDQ (10 μg/L) in wild-type nematodes, a decrease in *dbt-1* and *ivd-1* expressions was inhibited by treatment with leucine (5 mM) (Figure 6A). Meanwhile, after exposure to 6-PPDQ (10 μg/L) in wild-type nematodes, a reduction in NADH and FADH_2_ contents was suppressed through treatment with leucine (5 mM) (Figure 6B,C). Moreover, 6-PPDQ (10 μg/L)-induced mitochondrial dysfunction was inhibited through treatment with leucine (5 mM) in wild-type nematodes (Figure 6D,E). Additionally, 6-PPDQ (10 μg/L)-induced decreases in the expressions of *gas-1* and *mev-1* were further suppressed through treatment with leucine (5 mM) in wild-type nematodes (Figure 6F).

### 3.8. Beneficial Effect of Leucine Treatment Against Other Aspects of 6-PPDQ Toxicity

**Figure 7:**
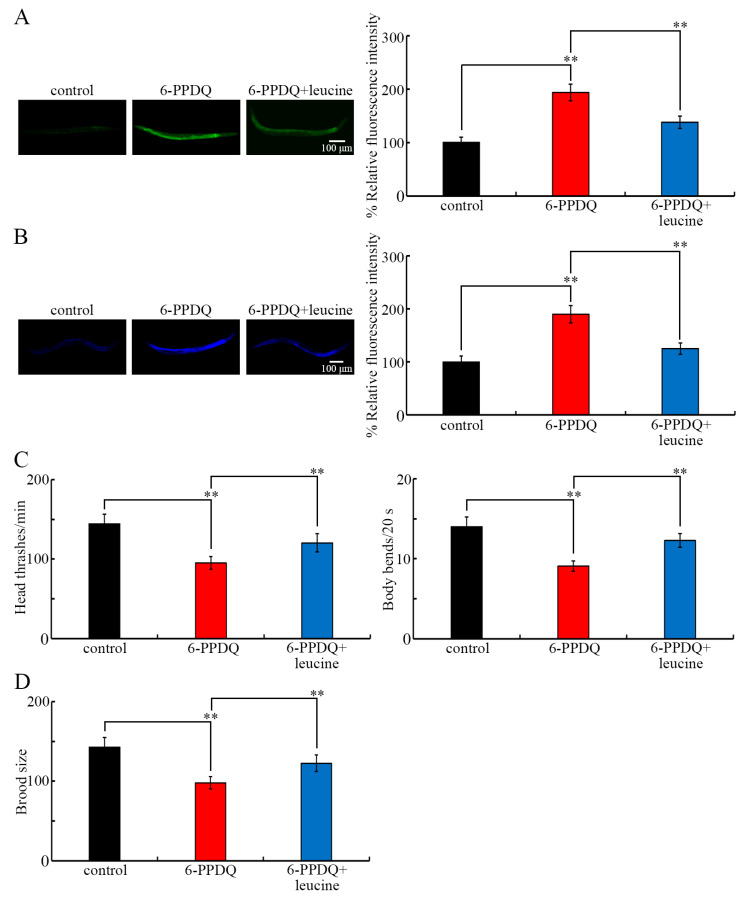
Effect of treatment with leucine (5 mM) on toxicity of 6-PPDQ (10 μg/L) in inducing intestinal ROS generation (**A**), causing intestinal lipofuscin accumulation (**B**), decreasing locomotion (**C**), and reducing brood size (**D**). ** *p* < 0.01.

After exposure to 6-PPDQ (10 μg/L) in wild-type nematodes, induced intestinal toxicity, reflected by intestinal ROS generation and lipofuscin accumulation, could be inhibited through treatment with leucine (5 mM) (Figure 7A,B). 6-PPDQ (10 μg/L)-caused locomotion inhibition was suppressed through treatment with leucine (5 mM) (Figure 7C) in wild-type nematodes. Moreover, the 6-PPDQ (10 μg/L)-induced reduction in brood size was inhibited following treatment with leucine (5 mM) in wild-type nematodes (Figure 7D).

## 4. Discussion

Leucine plays important modulatory functions in organisms [57,58]. Largely due to this, leucine supplementation has been frequently suggested in clinical settings for the treatment of some diseases [59,60,61]. In the current study, we observed s reduction in leucine content after exposure to 6-PPDQ at ERCs (Figure 1B). Besides the leucine content, the glutamate content could also be reduced by 6-PPDQ at ERCs [62]. Moreover, after exposure to 6-PPDQ at ERCs, the accumulation of glycogen, glucose, and lipid could be observed [33,34,46]. The transgenerational accumulation of lipid and glucose could be observed after exposure to 6-PPDQ at ERCs at the parental generation [32,46]. Therefore, exposure to 6-PPDQ at ERCs potentially disrupts multiple metabolic processes in organisms.

One of the biochemical bases for the observed reduction in leucine content was the inhibition of leucine adsorption. 6-PPDQ at 0.1–10 μg/L decreased the expression of *aat-1* encoding the amino acid transmembrane transporter (Figure 1C). In 6-PPDQ-exposed nematodes, the leucine content could be reduced by *aat-1* RNAi (Figure 1D). That is, 6-PPDQ could reduce the leucine content partially by inhibiting leucine adsorption through the suppression of AAT-1 expression. In *C. elegans*, AAT-1 was identified as one of the amino acid transporters and was shown to have the function to facilitate amino acid transport [63].

Another biochemical basis for the observed reduction in leucine content was the acceleration of leucine catabolism. 6-PPDQ at 0.1–10 μg/L increased the expression of *bcat-1* encoding the branched-chain amino acid transferase (Figure 2A). Meanwhile, in 6-PPDQ-exposed nematodes, the leucine content was increased by *bcat-1* RNAi (Figure 2B). Therefore, 6-PPDQ could also reduce leucine content by accelerating leucine catabolism through the activation of BCAT-1. In nematodes, BCAT-1 governs branched-chain amino acid metabolism [41] and is involved in the control of several other biological processes, such as the reprograming of proteasomal degradation and longevity [64].

In 6-PPDQ-exposed nematodes, accompanied with an increase in *bcat-1* expression, a decrease in expression of *dbt-1* and *ivd-1* encoding dihydrolipoamide branched-chain transacylase E2 and isovaleryl-CoA dehydrogenase was observed (Figure 2A), which suggests the inhibition in generation of NADH and FADH_2_ by 6-PPDQ through the influence on branched-chain amino acid metabolism, such as the leucine catabolism. Several lines of evidence support this. One is the observation of the reduction in NADH and FADH_2_ contents after exposure to 0.1–10 μg/L of 6-PPDQ (Figure 3E). Secondly, after 6-PPDQ exposure, the NADH content was reduced by the RNAi of *dbt-1* (Figure 3B), and the FADH_2_ content was reduced by the RNAi of *ivd-1* (Figure 3C). Thirdly, after 6-PPDQ exposure, NADH and FADH_2_ contents could be increased by the RNAi of *bcat-1* (Figure 2C,D). Fourthly, in 6-PPDQ-exposed nematodes, expressions of *dbt-1* and *ivd-1* could be increased by the RNAi of *bcat-1* (Figure 3D). Recently, we observed that 6-PPDQ could reduce NADH content by disrupting the citric acid cycle [48]. Therefore, 6-PPDQ at ERCs potentially reduced NADH and/or FADH_2_ contents by disrupting branched-chain amino acid metabolism and the citric acid cycle, simultaneously. *dbt-1* was also identified as the response gene to arsenic exposure in nematodes [65].

In organisms, NADH and FADH_2_ are two starting points for the electron transport chain, and the activities of mitochondrial complexes I and II could be inhibited by 6-PPDQ [35]. Moreover, the alteration in *bcat-1*, *dbt-1*, and *ivd-1* expressions was related to the induction of mitochondrial dysfunction in 6-PPDQ-exposed nematodes. Based on the analysis of endpoints of the oxygen consumption rate and ATP content reflecting mitochondrial function, the 6-PPDQ-induced increase in the oxygen consumption rate and the decrease in the ATP content could be inhibited by *bcat-1* RNAi and enhanced by *dbt-1* and *ivd-1* RNAi (Figure 4A,B). Meanwhile, we further observed that 6-PPDQ caused the induction of intestinal ROS generation and lipofuscin accumulation, an inhibition in locomotion, and a reduction in brood size could be suppressed by *bcat-1* RNAi and strengthened by the RNAi of *aat-1*, *dbt-1*, and *ivd-1* (Figure 5A–D). Therefore, the RNAi of *bcat-1* could cause resistance to 6-PPDQ toxicity, and the RNAi of *aat-1*, *dbt-1*, and/or *ivd-1* could induce susceptibility to 6-PPDQ toxicity, including the induction of mitochondrial dysfunction. These observations suggested that BCAT-1 had a different function from that of DBT-1 and IVD-1 in modulating biological processes, including mitochondrial function in 6-PPDQ-exposed nematodes. In nematodes, expressions of *gas-1* and *mev-1* encoding components of complexes I and II were decreased by 6-PPDQ, and the RNAi of these two genes caused susceptibility to 6-PPDQ toxicity on mitochondrial function [35]. Moreover, in 6-PPDQ-exposed nematodes, expressions of *gas-1* and *mev-1* were increased by *bcat-1* RNAi and decreased by *dbt-1* and *ivd-1* RNAi (Figure 4C). Additionally, in 6-PPDQ-exposed nematodes, expressions of *gas-1* and *mev-1* could also be decreased by the RNAi of *cts-1*, *idh-2*, *dlst-1*, and *dld-1*, which govern the citric acid cycle [48]. These observations suggest that both leucine catabolism-related enzyme genes and citric acid cycle-related enzyme genes could be involved in the control of mitochondrial function in 6-PPDQ-exposed nematodes by affecting the expressions of *gas-1* and *mev-1*.

Pharmacological treatment with leucine further demonstrated the beneficial effect of leucine treatment against 6-PPDQ toxicity, including the induction of mitochondrial dysfunction. Several lines of evidence indicated the beneficial effect of leucine treatment against 6-PPDQ damage on mitochondrial function. Firstly, the 6-PPDQ-induced increase in the oxygen consumption rate and the decrease in ATP content could be inhibited by leucine treatment (Figure 6D,E). Secondly, the 6-PPDQ-caused reduction in NADH and FADH_2_ contents could be suppressed by leucine treatment (Figure 6B,C). Thirdly, the 6-PPDQ-induced decrease in *gas-1* and *mev-1* expressions could be inhibited by leucine treatment (Figure 6F). Moreover, the 6-PPDQ-caused decrease in *dbt-1* and *ivd-1* expressions could also be suppressed by leucine treatment (Figure 6A), which suggests that leucine treatment potentially increased *gas-1* and *mev-1* expressions and mitochondrial function by activating *dbt-1* and *ivd-1* expressions. Besides the beneficial effect on mitochondrial function, leucine treatment could further inhibit the induction of intestinal ROS generation and lipofuscin accumulation (Figure 7A,B), accelerate locomotion (Figure 7C), and increase brood size (Figure 7D) in 6-PPDQ-exposed nematodes, which suggests the multiple possible beneficial effects of leucine treatment. In nematodes, treatment with glutamate could inhibit 6-PPDQ by activating the corresponding receptors of GLR-1, GLR-2, and GLR-4 [62]. In zebrafish larvae, ghrelin, a growth hormone secretagogue receptor (GHSR) agonist, also showed neuroprotective function against 6-PPDQ toxicity [66]. That is, 6-PPDQ toxicity can be inhibited by different pharmacological treatments through different mechanisms.

## 5. Conclusions

Together, we observed a reduction in leucine content by 6-PPDQ at ERCs in nematodes. This leucine reduction in 6-PPDQ-exposed nematodes was associated with an inhibition in leucine adsorption, indicated by a decrease in *aat-1* expression. Additionally, this leucine reduction in 6-PPDQ-exposed nematodes was related to an increase in leucine catabolism, indicated by an increase in *bcat-1* expression. Additionally, in 6-PPDQ-exposed nematodes, expressions of *dbt-1* and *ivd-1* were decreased, which was linked to a reduction in NADH and FADH_2_ contents and mitochondrial dysfunction. Pharmacological treatment with leucine could inhibit the decrease in *dbt-1* and *ivd-1* expressions, the reduction in NADH and FADH_2_ contents, and the mitochondrial dysfunction caused by 6-PPDQ exposure. Our results provide new insight into the induction of mitochondrial dysfunction by 6-PPDQ exposure at ERCs in organisms.

## Data Availability

The original data presented in this study are included in the article/Supplementary Materials; further inquiries can be directed to the corresponding author.

## Supplementary Materials

The following supporting information can be downloaded at https://www.mdpi.com/article/10.3390/toxics13070544/s1. Figure S1: RNAi efficiency of aat-1, bcat-1, ivd-1, and dbt-1. ** *p* < 0.01 vs wild-type (L4440); Table S1: Primer information for qRT-PCR.
