# Supplementary material for: Exposure to 6-PPD Quinone Disrupts Adsorption and Catabolism of Leucine and Causes Mitochondrial Dysfunction in Caenorhabditis elegans
Source: Toxics. 2025 Jun 28;13(7):544. doi: 10.3390/toxics13070544 (PMC12300368; doi:10.3390/toxics13070544)
Supplement: Supplementary file 1 [file toxics-13-00544-s001.zip › toxics-3708330-supplementary.pdf]

Supporting Information:

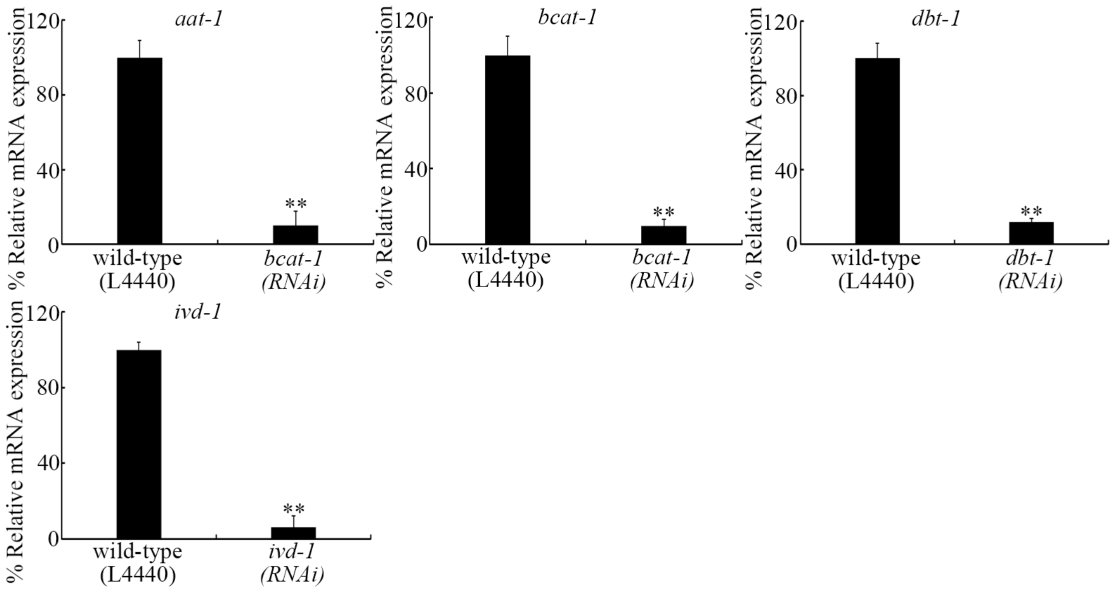

Fig. S1 RNAi efficiency of *aat-1*, *bcat-1*, *ivd-1*, and *dbt-1*. $^{**}P < 0.01$ *vs* wild-type (L4440).

Table S1. Primer information for qRT-PCR

Gene	Forward primer (5'-3')	Reverse primer (5'-3')
aat-1	ACGAAACCTTCCACTCGCAA	GGCATTTGTCCTTCACGAGC
bcat-1	GATTGGGATGCCGAGAGAGG	TTCTGGACGGAACATGCGAA
dbt-1	GCCTCTCCAGTTGTCTTCCC	TGCTGAAGCGAGCCATTGTA
ivd-1	CGGATCATTCCAGCTCCTCC	AATCACACCGGCACAATCCT
gas-1	TCTCAACTTCGGACCACAGC	CAACAGCCAAAGACCAAGCC
mev-1	TTTGCCGTTCGATTTCACCG	AAAGAAGGCGGAGCATCTGT
tba-1	TCAACACTGCCATCGCCGCC	TCCAAGCGAGACCAGGCTTCAG
